# Stimulating meditation: a pre-registered randomised controlled experiment combining a single dose of the cognitive enhancer, modafinil, with brief mindfulness training

**DOI:** 10.1177/0269881121991835

**Published:** 2021-03-01

**Authors:** Emily M Thomas, Tom P Freeman, Patrick Poplutz, Kane Howden, Chandni Hindocha, Michael Bloomfield, Sunjeev K Kamboj

**Affiliations:** 1 Clinical Psychopharmacology Unit, University College London, London, UK; 2 Addiction and Mental Health Group (AIM), University of Bath, Bath, UK; 3 Translational Psychiatry Research Group, University College London, London, UK

**Keywords:** Mindfulness, cognitive enhancement, modafinil, attention, mind wandering

## Abstract

**Background:**

Mindfulness-meditation has a variety of benefits on well-being. However, individuals with primary attentional impairments (e.g. attention deficit disorder) or attentional symptoms secondary to anxiety, depression or addiction, may be less likely to benefit, and require additional mindfulness-augmenting strategies.

**Aims:**

To determine whether a single dose of the cognitive enhancer, modafinil, acutely increases subjective and behavioural indices of mindfulness, and augments brief mindfulness training.

**Methods:**

A randomised, double-blind, placebo-controlled, 2 (drug: placebo, modafinil) × 2 (strategy: mindfulness, relaxation control) experiment was conducted. Seventy-nine meditation-naïve participants were assigned to: placebo–relaxation, placebo–mindfulness, modafinil–relaxation or modafinil–mindfulness. Pre-drug, post-drug and post-strategy state mindfulness, affect and autonomic activity, along with post-strategy sustained attention and mind-wandering were assessed within a single lab session. After the session, participants were instructed to practice their assigned behavioural strategy daily for one week, with no further drug administration, after which, follow-up measures were taken.

**Results:**

As predicted, modafinil acutely increased state mindfulness and improved sustained attention. Differential acute strategy effects were found following mindfulness on autonomic activity but not state mindfulness. There were no strategy or drug effects on mind-wandering. However, exploratory analyses indicated that participants receiving modafinil engaged in more strategy practice across strategy conditions during follow-up.

**Conclusions:**

Modafinil acutely mimicked the effects of brief mindfulness training on state mindfulness but did not enhance the effects of this training. Limitations of the current study, and recommendations for future research examining modafinil as an adjunct to mindfulness- (or relaxation-) based treatments are discussed.

## Introduction

Mindfulness is a meditation practice derived from the Buddhist contemplative tradition and can be viewed as a form of cognitive (attentional) training. Although definitions of mindfulness vary and emphasise different underlying psychological processes (e.g. experiential acceptance, interoceptive discernment, insight, compassion), most definitions acknowledge the centrality of attention-regulation as a foundational skill (Bishop et al., 2004; Kabat-Zinn, 1994). Neuropsychological definitions of attention include a capacity to sustain, switch and inhibit the allocation of information processing resources to a particular internal or external object (Posner and Petersen, 1990). An exemplar of an internal object of attention is *the breath*, and mindful breathing is a prototypical mindfulness exercise intended to increase attention-regulation through repeated practice. This typically involves a combination of an extended singular focus on the breath (sustained and focused attention), cognitive control of unrelated thoughts (executive function/inhibition) and refocusing of attention to the breath when the mind wanders (attentional switching). Although novices often struggle with mindfulness training exercises, and typically experience frequent ‘task-unrelated thoughts’ (mind-wandering), long-term training is associated with reduced mind-wandering (Mrazek et al., 2012) and enhanced performance on attentional tasks (Lutz et al., 2008). These behavioural changes are thought to reflect enduring structural and functional alterations in brain regions that, for example, subserve interoceptive attention (Farb et al., 2013).

Mindfulness meditation is increasingly used as a component of psychological treatments for a variety of mental and physical health problems. Clinical trials of mindfulness-based interventions (MBIs) have demonstrated the strongest evidence for preventing depressive relapse, although MBIs also show promise for other indications (Goldberg et al., 2018). However, in addition to clinical trials that have examined this typical, extended format of mindfulness training, single-session experimental studies of very brief training (single session ‘mindfulness induction’: typically ⩽ 20 min) have been conducted in healthy volunteers. Although some such studies demonstrated immediate benefits of brief mindfulness training exercises (e.g. on negative affect: Schumer et al., 2018), such studies are typically mechanistic, rather than aiming to establish clinical efficacy. For example, studies of mindfulness inductions have examined the neurophysiological basis of mindful mental states and the association between such states (or their accompanying neurophysiological changes), and adaptive changes in affect, cognition and behaviour; others have sought to identify active components of more comprehensive treatments by examining isolated mindfulness techniques (Gill et al, 2020; Schumer et al, 2018). Given the brevity of these experimental mindfulness trainings/inductions, it is perhaps unsurprising that the observed effects are typically small (*d* ≈ 0.2) and short-lived (Gill et al, 2020; Schumer et al., 2018). Despite this, such experimental studies are potentially highly informative in terms of identifying characteristics of participants who might show early treatment gains during a comprehensive/extended MBI, or in terms of developing novel, brief MBIs.

The nature of typical MBIs, both in terms of their short-term demands (prolonged within-session practice), and their relatively extended duration (typically spanning *c*. eight weeks), may not be optimal for some patient groups, and abbreviated treatments might be more accessible to a wider range of patients. However, other obstacles can prevent *initial engagement* with MBIs. Indeed, the specific focus of MBIs on self-regulation of attention represents a potential barrier in patients for whom attentional and motivational difficulties are an inherent symptom of their disorder (Barnhofer et al, 2009; Eysenck et al, 2007). One approach to overcoming such obstacles may be to employ biological strategies (e.g. non-invasive brain stimulation or cognitive enhancing drugs) to support the basic attentional capacities required during MBIs. Laboratory-based studies of brief mindfulness trainings are ideally placed to preliminarily test such MBI-adjunctive treatments.

The wakefulness-promoting drug, modafinil, which has cognitive- (especially attention-) enhancing properties, may be an example of such an adjunctive treatment. Although the molecular-pharmacological basis of its wakefulness promoting and pro-cognitive effects remain unclear, a range of experimental approaches indicate that modafinil occupies dopamine and noradrenaline transporters (e.g. Madras et al, 2006), suggesting one potential molecular mechanism of action for its clinical effects (Thorpy and Bogan, 2020). Behaviourally, the effects of modafinil (improvements in executive functioning, attention, learning and memory; Battleday and Brem, 2015) seem to resemble improvements in cognition that have been described in some studies of brief mindfulness training (see Gill et al., 2020). Given this resemblance, modafinil might also acutely *mimic* subjective aspects of the attentional state(s) attained through brief mindfulness training and/or acutely *augment* the effects of such training.

As a first step to determining whether pharmacological augmentation of mindfulness training might be a clinically viable strategy, the current laboratory-based study used a randomised double-blind, placebo controlled design to examine the *acute* effects of a brief mindfulness induction combined with a single dose of modafinil. We employed a parsimonious mixed within-between subjects design in which assessment of pharmacological effects were separated from those of a behavioural manipulation, while also allowing their combined effects to be tested (e.g. Kamboj et al., 2015, 2018). Our pre-registered hypotheses were that, compared to an active relaxation control, brief mindfulness training would increase state mindfulness, improve sustained attention and decrease mind wandering. We also predicted that compared to placebo, modafinil would have similar directional effects to brief mindfulness training on these outcomes. Finally, we examined the possibility of additive or synergistic effects between mindfulness and modafinil by testing strategy × drug interactions.

## Methods

The study received ethical approval from the University College London research ethics committee. Hypotheses, methods and analysis plan were pre-registered on the Open Science Framework (OSF; https://osf.io/34xn9).

### Study design

We employed a randomised, double blind 2 (drug) × 2 (strategy), factorial, between-subjects design with additional within-subjects factors of time (for acute effects) or day (for effects at one week follow-up; see below). Both a matched placebo drug control and a well-matched active control for mindfulness (i.e. relaxation) were used. This resulted in four experimental conditions: placebo–relaxation, modafinil–relaxation, placebo–mindfulness and modafinil–mindfulness. In all groups, subjective effects were tested before drug, after drug and after the mindfulness or relaxation strategy, while the drug was still expected to be active (i.e. the combined effects of strategy and drug). Behavioural outcomes were only tested after the strategy.

### Participants

Written informed consent was obtained from participants prior to any experimental procedure. Eighty healthy, meditation naïve participants (i.e. with < 20 h lifetime mediation experience) were recruited from the university and local community through online and paper-based adverts and were randomly and evenly (*n* = 20/group) assigned to placebo–relaxation, modafinil–relaxation, placebo–mindfulness, modafinil–mindfulness, with equal numbers of men (*n* = 10) and women (*n* = 10) per cell. Of the original sample, two participants’ data could not be included (see Supplemental material). One of these participants was replaced, leaving a final cohort of 79. All were compensated for their time (£25). See Supplemental material for further details on screening, inclusion/exclusion criteria, randomisation and sample size determination.

Participants were blind to drug condition and were not aware that there were two strategy conditions; nor were the terms ‘relaxation’ or ‘mindfulness’ used in any study material. Experimenters were blind to both drug and strategy condition.

### Procedure

**Figure 1.**
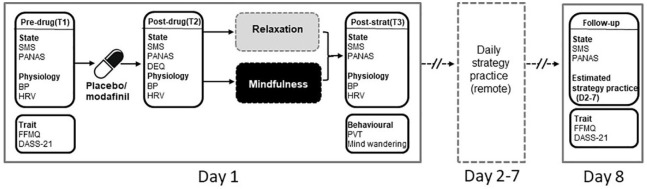
Outline of experimental procedure for the experimental session (Day 1), remote self-practice (Days 2–7) and follow up (Day 8). Day 1 consisted of three time points: pre-drug (T1), post-drug (T2) and post-strategy (T3). SMS, State Mindfulness Scale; PANAS, Positive And Negative Affect Schedule; FFMQ, Five Facet Mindfulness Questionnaire; DASS-21, Depression, Anxiety and Stress Scale (21 item); DEQ, Drug Effects Questionnaire (T2 only); PVT, psychomotor vigilance task; BP, blood pressure; HRV, heart rate variability.

Tasks and measures completed during the lab session (‘Day 1’) are outlined in Figure 1. Briefly, after consenting and providing basic demographics, participants were fitted with an ECG device and completed a series of self-report questionnaires, items from which were presented, and responses recorded using the online survey program, Qualtrics (Provo, UT). They completed pre-drug (time point 1: T1) state measures of mindfulness (State Mindfulness Scale (SMS); Tanay and Bernstein, 2013) and positive and negative affect (Positive and Negative Affect Schedule (PANAS); Watson et al., 1988). They also completed the brief (15 item) Five Facet Mindfulness Questionnaire (FFMQ; Baer et al., 2006) and the Depression, Anxiety and Stress Scale (DASS-21; Lovibond and Lovibond, 1995), which respectively assessed baseline levels of trait mindfulness and general mental wellbeing (see Supplemental material for more details on self-report measures). Blood pressure (BP) was also assessed at T1, and heart rate (variability) (HRV) was sampled continuously (see section below on HRV for details on sampling periods).

Participants then swallowed capsules containing either modafinil or placebo with water, followed by a 2 h rest/drug absorption period, during which they either watched low arousal nature documentaries, relaxed or read. After this, participants provided a BP reading and completed the post-drug (time point 2: T2; see Figure 1) questionnaires, which in addition to the SMS and PANAS, included the Drug Effects Questionnaire (DEQ; Morean et al., 2013), which was used to gauge the general strength of subjective drug effects, along with rewarding, aversive and motivational effects. Participants then underwent their assigned strategy training (mindfulness induction or relaxation), followed by the mind-wandering task, post-strategy state questionnaires (time point 3: T3), a final BP reading and the psychomotor vigilance task (PVT). Before leaving the laboratory session, participants were instructed to independently practice their assigned strategy daily until follow-up (Days 2–7; Figure 1) and were provided with a reminder flashcard as a prompt to practice. A remote follow-up assessment was completed after a week (Day 8), which included the SMS, PANAS, FFMQ and DASS-21.

### Drugs

Identical opaque gelatine capsules containing either modafinil (200 mg plus additional milk powder filling) or placebo (milk powder alone) were administered with water (see Supplement material for details on drug preparation). Integrity of double-blinding was tested by eliciting independent treatment guesses from participant and researcher, who entered their guess (placebo or modafinil) directly onto the Qualtrics survey page at the end of the experimental session, such that neither was aware of the other’s response.

### Strategy instructions

Mindfulness and relaxation training instructions were written/adapted from existing material by author SKK, from whom full instructions (scripts or audio recordings) are available. Further details on instruction development and validation are provided in the Supplemental material. The audio recordings of the strategy instructions were manually deployed by the experimenter according the randomisation code via Qualtrics, and presented to participants through headphones. Strategy allocation was concealed from the experimenter, as the audio recordings were only identified with the labels ‘recording A’ and ‘recording B’ on Qualtrics and the same pre-set audio level was used across all participants to ensure there was no audio leakage through headphones. The strategy instructions were presented in two segments, the total duration of which was < 10 min for each strategy. The first segment was an explanation of the strategy and a brief practice, after which credibility and expectancy were assessed (see below). This was followed by the second segment, which was the main strategy training.

*Mindfulness training*: mindfulness instructions were based on previously published material from a variety of sources (e.g. Williams and Penman, 2011). They were designed to be brief and widely comprehensible and were intended primarily to promote attention to the breath through repeated encouragement to ‘notice’ the experience of breathing, and to return to this ‘noticing’ if the participant became aware that their attention had drifted. Such instructions are consistent with focused attention-type meditation (Lutz et al., 2008) which is commonly used as a preparatory component of mindfulness meditations. The instructions deliberately avoided the use of the term ‘mindfulness’ to conceal the overt aims of the strategy and reduce expectancy effects (Kamboj et al., 2017), and instead referred to this strategy as ‘focused breathing’ throughout the experiment.

*Relaxation training*: the relaxation strategy instructions were designed to closely match the wording, duration and complexity of language (reading level) of the mindfulness instructions. This strategy encouraged breath *control*, through deep regulated breathing, along with muscle relaxation (the strategy was referred to as ‘regulated abdominal breathing’ in the audio recording). As with the mindfulness strategy, participants were instructed to return to performing regulated breathing if they noticed their attention had drifted.

At the end of the lab session on Day 1, participants were instructed to ‘practice the breathing exercise that you learned in this session for 10 min every day for the next week [i.e. Day 2–7])’. They were also given a credit-card sized flashcard to carry with them as a reminder to practice. Since experimenters were blind to strategy condition, no further instructions were provided by the experimenter on use of the assigned strategy during follow-up. No other prompts, reminders or encouragement were given to participants in relation to strategy practice and there was no daily monitoring of practice.

### Behavioural and physiological measures

#### Mind-wandering task

Mind-wandering – which involves a shift in attention to task-unrelated thoughts – may be a cognitive marker of (lack of) mindfulness. We assessed mind-wandering using an adapted version of the task described by Mrazek et al. (2012), which captures ‘probe-caught’ and ‘self-caught’ instances of mind-wandering. During the task, which was performed only once, during the post-strategy (T3) phase, participants were instructed to perform the mindfulness or relaxation strategy that they had just learned, and to make a mouse click if their mind wandered from the strategy. Both self-caught (mouse clicks in the absence of a probe) and probe-caught (mouse clicks within 1 s of an audio probe) instances were recorded. Probes were presented with a pseudo-random inter-probe interval of 40-80 s (average interval 60 s), and a total of 15 probes were presented during the 16-min task.

#### PVT

The PTV is a reaction time-based measure of sustained attention, performance on which is sensitive to both modafinil (Dinges and Weaver, 2003) and mindfulness training (Kaul et al., 2010; Wong et al., 2018). The version of the task used here (PC-PVT 2.0) is described in detail in Khitrov et al. (2014). Briefly participants were instructed to respond as quickly as possible to a visual stimulus (a reaction time counter displayed in milliseconds) presented at random intervals on the computer screen. As recommended (Khitrov et al., 2014), responses were recorded via a high-performance gaming mouse (Harpoon RGB gaming mouse, Corsair) to ensure measurement accuracy. Like the mind-wandering task, the PVT was performed once, at the post-strategy (T3) time point (see Figure 1).

#### BP and HRV

BP was assessed at baseline/pre-drug (T1), post-drug (T2), and post-strategy (T3) using a commercial wrist-worn BP monitor. As a physiological measure of strategy (and drug) effects, we used HRV which is thought to reflect activity of brain networks involved in emotion regulation (Mather and Thayer, 2018) and has been proposed as a biomarker of mindful mental states (Krygier et al., 2013). A Firstbeat ECG device (Bodyguard 2, Firstbeat Technologies) was attached with two electrodes (below the right clavicle and left ribcage) at the beginning of the testing session, which recorded inter-beat (RR) intervals throughout the testing session. HRV parameters were extracted offline with the Kubios software package (Tarvainen et al., 2014) using 5-min segments of RR data at T1, T2 and T3 (T2 and T3 HRV corresponded to the first 5-min after the 2 h drug absorption period and the final 5-min of strategy instruction respectively). As in our previous study (Kamboj et al., 2017), the root mean square of successive differences (RMSSD) was used to index HRV.

### Control measures

Devilly and Borkovec’s (2000) questionnaire was adapted to assess strategy credibility and expectancy after the explanation/brief practice segment of the recorded strategy instructions. An average credibility rating was based on three questions about how logical, successful and recommendable each strategy was in relation to ‘[helping] you to calm your mind’. Expectancy was based on a single question relating to how much the strategy was felt to be potentially helpful. Each expectancy and credibility question was rated on a 1 (‘not at all’) to 9 (‘very much’) scale. As an additional control measure, participants completed a manipulation check (Arche and Craske, 2006) at the end of the study, indicating whether they closely followed the audio instructions for the strategy (0 = very untrue; 7 = very true).

### Follow up

Participants completed an online follow-up assessment that included the FFMQ, DASS-21, SMS and PANAS. They also provided estimates of the number of occasions that they practiced their assigned strategy and the number of minutes of practice per occasion during Days 2–7. A total practice time for the follow-up was derived from these estimates (occasions × minutes/occasion).

### Statistical analyses

Statistical analysis was conducted using SPSS (version 25; IBM). Details on data handling are provided in the Supplemental material. For all analyses, an alpha threshold of 0.05 was used and two-tailed tests are reported throughout. All post-hoc pairwise tests of significant ANOVA effects were Bonferroni corrected. The analysis plan was pre-registered on the Open Science Framework (https://osf.io/34xn9) although minor departures from this were required because of an incorrect specification of the role of the two between-subjects factors. Thus, instead of a single between-subjects factor, ‘Group’, with four levels, the primary analyses (should have) actually involved *two* between-subjects factors, namely strategy (mindfulness; relaxation) and drug (placebo; modafinil). Within-subjects factors of Time (T1, T2 and T3) and Day (Day 1 and Day 8) were used in repeated measures analyses.

Outcomes assessed at a single time point (mind-wandering, PVT, amount of strategy practice) were analysed using univariate factorial (strategy × drug) ANOVA or independent samples *t*-test (strategy compliance ratings; DEQ scores) with false discovery rate adjustment as appropriate. Continuous variables measured at the pre-drug, post-drug and post-strategy time points on Day 1 (state mindfulness, HRV and affect) were analysed using three-way repeated measures ANOVAs, with Time (T1, T2, T3) as the within-subjects factor. Day (Day 1, 8) was the within-subjects factor when assessing ‘long-term’ effects on SMS and PANAS (measured at T1) and DASS-21 and FFMQ. Where Mauchly’s test indicated significant departures from sphericity, *dfs* and *p* values were adjusted using Greenhouse–Geisser correction (reflected in non-integer *dfs* accompanying some *F* statistics). Note, no a priori predictions were made for drug and/or strategy effects on affect (PANAS), HRV or any of the longer-term effects (including amount of practice). Results from these analyses should therefore be considered exploratory.

Omnibus effect sizes are reported as (partial) eta squared values; effect sizes associated with post-hoc comparisons are expressed as Cohen’s *d*, with appropriate correction for repeated measures correlations for within-subjects comparisons across two time-points (Dunlap et al., 1996). Means ± standard deviations (SD) are reported in the text and tables, and means ± standard errors (SE) are indicated in Figures.

## Results

### Participants

Baseline demographic and ‘trait’ measures are summarised in Table 1. The values are within the ranges expected of a normative sample.

**Table 1.** Means and standard deviations for baseline measures.

	Placebo		Modafinil	
	Relax^a^	Mindful	Relax	Mindful
**Age**	26.68 (7.19)	24.35 (5.39)	23.80 (3.71)	26.20 (5.60)
**DASS (Dep.)**	8.11 (5.10)	6.90 (5.13)	7.00 (6.10)	8.80 (6.72)
**DASS (Anx.)**	5.47 (4.89)	4.80 (3.27)	6.20 (5.06)	7.16 (5.47)
**DASS (Stress)**	9.37 (4.81)	9.20 (4.27)	12.20 (5.80)	13.60 (7.21)
**FFMQ**	46.95 (7.01)	51.05 (5.70)	50.50 (6.93)	49.60 (5.63)

a *n* = 19 (nine men, 10 women; other groups *n* = 20, 10 men, 10 women).

FFMQ, Five Facet Mindfulness Questionnaire; DASS, 21-item Depression, Anxiety and Stress Scale.

### Subjective state measures

#### State mindfulness

**Figure 2.**
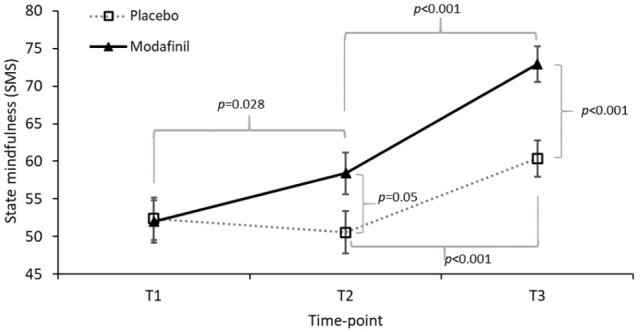
Estimated marginal means (± SE) for drug × time effects on state mindfulness (State Mindfulness Scale; SMS). T1, pre-drug; T2, post-drug (pre-strategy); T3, post-strategy. All significant pairwise comparisons are indicated; associated *p* values are Bonferroni corrected.

No main effect or interactions involving strategy were found (*p* values ⩾ 0.125), indicating that, contrary to our hypothesis, the mindfulness strategy did not produce the intended larger increase in state mindfulness relative to relaxation. However, as shown in Figure 2 there was a time × drug interaction: *F*(1.8,137.0) = 6.268, *p=* 0.003, *η*_p_^2^ = 0.077). Collapsing across strategy, pairwise Bonferroni corrected tests comparing state mindfulness at T1 and T2 separately for each of the drug conditions, showed no change following placebo (*p* > 0.99), but a significant, though modest, increase following modafinil (*p* = 0.030, *d* = 0.344), as predicted. The T2-to-T3 change in state mindfulness was significant and of moderate-large magnitude following placebo (collapsed across strategy; *p* < 0.001; *d* = 0.633) but was larger following modafinil (collapsed across strategy; *p* < 0.001, *d* = 0.821). Complementary post-hoc tests of drug effects at each level of time showed that state mindfulness was marginally higher at T2 in the modafinil versus placebo conditions (*p* = 0.053, *d* = 0.45), with the divergence between drug conditions being more pronounced at T3 (*p* < 0.001, *d* = 0.83). Collectively these results suggest that modafinil may enhance the effects of behavioural interventions that encourage either relaxation *or* mindfulness.

#### Positive and negative affect

**Figure 3.**
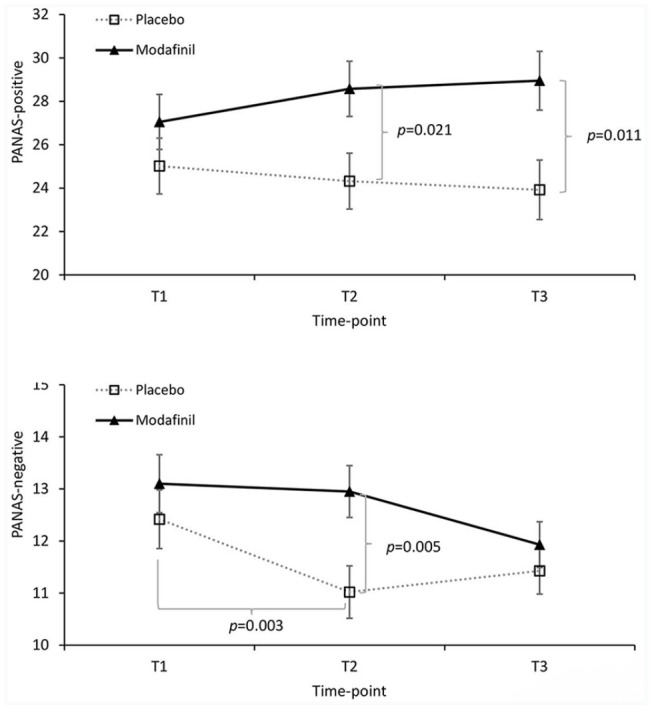
Drug × time effects for (a) positive affect (mean Positive and Negative Affect Schedule (PANAS)-positive affect scores ± SE) and (b) negative affect (untransformed mean PANAS-negative ± SE are presented but analyses were performed on log transformed data because of skewness; see Supplemental material). T1, pre-drug; T2, post-drug (pre-strategy); T3, post-strategy. Significant pairwise comparisons are indicated with Bonferroni corrected *p* values.

There were no significant two or three-way interactions for PANAS-positive scores (*F* values ⩽ 2.263, *p* values ⩾ 0.083) and no main effect of strategy (*F*(1,75) = 0.195, *p* = 0.660). However, although the time × drug interaction (Figure 3(a)) did not reach significance (*F*(1.8, 131.7) = 2.623, *p* = 0.083), there was a main effect of drug (*F*(1,75) = 5.077, *p* = 0.027, *η*_p_^2^ = 0.063). Given this, and the presence of a marginal interaction, exploratory Bonferroni corrected pairwise comparisons were performed to explore the effect of drug at each level of time. As expected, there were no pre-drug differences between the placebo and modafinil conditions in positive affect (T1, *p* = 0.264). However, PANAS-positive scores were higher following modafinil compared to placebo at T2 (*p* = 0.021, *d* = 0.536) and T3 (*p* = 0.011, *d* = 0.595). To determine if change in positive affect had a different association with change in state mindfulness in the two drug conditions, correlations between ΔPANAS-positive (T2–T1) and ΔSMS (T2–T1) were performed for each drug. However, these did not suggest different associations in the two drug conditions (*r*_placebo_(37) *=* 0.562, *p* < 0.001; *r* _modafinil_ (38) *=* 442, *p* = 0.004). Additional bivariate correlations between ΔSMS and other outcomes are reported in the Supplemental material.

On the PANAS-negative subscale there were no interactions involving Strategy (*F* values ⩽ 2.425, *p* values ⩾ 0.092; note, analysis of PANAS-negative was performed on log transformed values; see Supplementary material on data handling). However, a main effect of time (F(2,150) = 5.736, *p* = 0.004, *η*_p_² = 0.113) was subsumed under a time × drug interaction (*F*(2,150) = 3.143, *p* = 0.046, *η*_p_² = 0.04; Figure 3(b)). Post-hoc tests of drug and time at each level of time and drug respectively showed a significant and moderate-sized reduction in negative affect between T1 and T2 in the placebo group (*p* = 0.003, *d* = −0.60), but no change in the modafinil group (*p* > 0.99). A small decrease (*d* = −0.291) between T2 and T3 in the modafinil group was not significant (*p* = 0.086), and there was no change between T2 and T3 in the placebo group (*p* > 0.99). Consistent with the pairwise time effects, pairwise drug effects showed that PANAS-negative scores were significantly higher following (at T2) modafinil versus placebo (*p* = 0.005, *d* = 0.646), but scores converged at T3 (*p* = 0.377; Figure 3(b)). These effects of modafinil on affect (higher positive *and* negative affect) are consistent with previous findings (Taneja et al, 2007).

### Physiological indices

**Figure 4.**
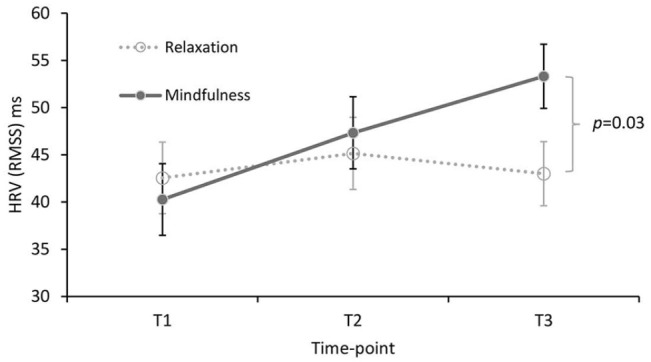
Strategy × time effect on heart rate variability (RMSSD; mean ± SE). T1, baseline/pre-drug; T2, post-drug (pre-strategy); T3, post-strategy. The *p* value for the post-hoc mindfulness versus relaxation comparison at T3 is Bonferroni corrected.

There was no main effect and no interactions involving drug on the RMSSD measure of HRV (*F* values ⩽ 1.776; *ps* ⩾ 0.173) However, a significant main effect of time (*F*(2,136) = 5.612, *p* = 0.005, *η*_p_^2^ = 0.076) was subsumed under a time × strategy interaction (*F*(2,136) = 4.743, *p* = 0.010, *η*_p_² = 0.065; Figure 4).

Since strategy instructions were given after T2, the focal effect of time was examined between T2 and T3 in each of the strategy conditions with post-hoc pairwise tests (Bonferroni corrected). The apparent increase in RMSSD in the mindfulness conditions (Figure 4) failed to reach significance (*p* = 0.074, *d* = 0.30); the T2–T3 change in the relaxation conditions was clearly non-significant (*p* > 0.99, *d* = − 0.121). However, the difference between relaxation and mindfulness at T3 was significant (*p* = 0.031, *d* = 0.519; Figure 4).

There was a modest upward trend in systolic BP across the Day 1 time-points (from *c.* 108 mmHg at T1 to *c.* 111 mmHg at T3; main effect of time *F*(2,138) = 3.237, *p* = 0.042). However, there were no effects involving strategy on systolic BP (*F* values ⩽ 0.520; *ps* ⩾ 0.596). Also, in line with previous research (Hou et al., 2005) modafinil had no effect (no main or interaction effects involving drug) on systolic BP (values *F* ⩽ 0.697, *ps* ⩾ 0.407) or heart rate (*F* values ⩽ 2.091, *ps* ⩾ 0.127).

### Behavioural measures

#### Sustained attention

**Figure 5.**
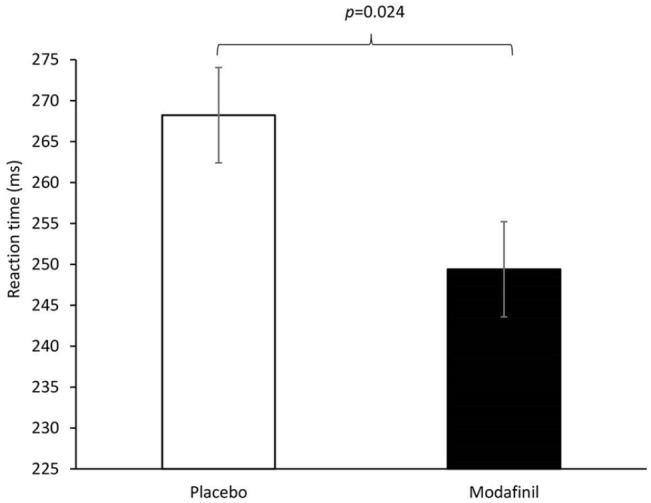
Mean reaction times (±SE) for the psychomotor vigilance task.

As predicted, a factorial drug × strategy ANOVA showed a main effect of drug on PVT reaction times (*F*(1,70) = 5.234, *p* = 0.025, *η*² = 0.07). As illustrated in Figure 5, this reflected faster reaction times in the modafinil relative to placebo group. There was no drug effect on the mean number of valid responses (placebo mean ± SD: 92.76 ± 5.05; modafinil: 93.78 ± 3.18), suggesting that the effect on reaction times did not reflect a speed-accuracy trade-off (*t*(72) = 1.05, *p* = 0.298). There was no drug × strategy interaction (*F*(1,70) = 0.017, *p* = 0.897) and the effect of strategy did not reach statistical significance (*F*(1,70) = 3.175, *p* = 0.079). Descriptively however, the relaxation conditions had marginally faster reaction times (mean ± SD: 251.5 ± 36.2 ms) than the mindfulness conditions (266.1 ± 36.2 ms).

#### Mind-wandering task

For self-caught mind-wandering events (mean ± SD collapsed across drug and strategy conditions: 4.18 ± 2.89) and probe-caught events (across conditions: 16.81 ± 13.32), contrary to pre-specified hypotheses, there were no main effects of strategy or drug and no two or three-way interactions involving drug or strategy (all *F* values ⩽ 3.034, *p* values > 0.086).

### Follow up: tests of enduring effects at Day 8

**Figure 6.**
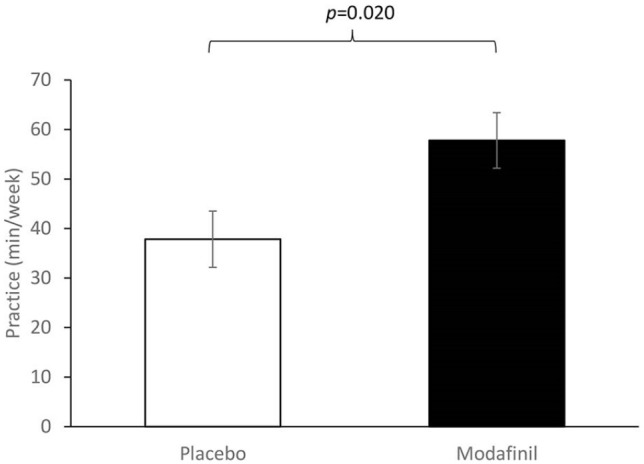
Amount of strategy practice (in minutes) in the week following the lab session by drug group (mean ± SE).

The majority of participants (*n* = 73; 92.4%) completed the follow-up time-point on Day 8 and indicated that they engaged in at least some strategy practice during the follow-up period (⩾ 1 occasion). Post-hoc exploratory analysis showed that the drug × strategy effect on total minutes of practice during the follow-up period (Days 2-7) was not significant (*F*(1,75) = 0.251, *p* = 0.618) and the strategy main effect was also non-significant (*F*(1,75 ) = 0.354, *p* = 0.553). However, there was a significant main effect of drug (*F*(1,75) = 5.613, *p* = 0.020). During the follow-up, participants in the placebo groups reported practicing their assigned strategy for a total of 35.78 + 32.53 min (mean + SD) compared to 54.78 + 34.71 in the modafinil groups, representing 53% more time spent on strategy practice in participants who received modafinil on Day 1 (Figure 6).

There was a main effect of Day (Day 1 v. Day 8) on the SMS, with all four Drug-Strategy combinations showing an increase in state mindfulness (*F*(1,69) = 15.207, *p* < 0.001; see Supplemental Table S2). No day effects were found for PANAS positive or negative, DASS-depression or FFMQ (all day main effect *F* values ⩽ 1.366, *p* values ⩾ 0.290). Tests of interactions involving day and drug and/or strategy showed that there were no sustained drug or strategy effects at Day 8 on SMS, FFMQ, PANAS or DASS-depression (*F* values ⩽ 3.377, *p* values ⩾ 0.070).

### Control measures and manipulation checks

#### Expectancy, credibility and within-session strategy compliance

As expected for closely matched strategies, average credibility ratings for relaxation (mean ± SD: 6.21 ± 1.53) and mindfulness (6.54 ± 1.23) were not significantly different (*t*(77) = 1.050, *p* = 0.297). Similarly, expectancy was similar for relaxation (5.79 ± 1.45) and mindfulness (6.10 ± 1.45; *t*(77) = 0.935, *p* = 0.353). Compliance did not differ between relaxation (6.03 ± 1.06) and mindfulness (6.08 ± 1.05) strategies (*t*(77) = 0.208, *p* = 0.836).

#### Blinding and subjective drug effects

Twenty-eight participants (of *n* = 39) in the placebo groups correctly guessed their treatment (72%); in the modafinil group 17/40 (43%) guessed correctly (χ^2^(1, *n* = 79) = 1.764, *p* = 0.184). Researchers guessed at chance levels for placebo (59% correct) and modafinil (50% correct; χ^2^(1, *n* = 79) = 0.641, *p* = 0.423). Overall, this indicated that double blinding was preserved, although participants in the modafinil groups did indicate higher ratings on the ‘*feel*’ item of the DEQ (*p*_(FDR-adjusted)_ = 0.035; Table 2).

**Table 2.** Subjective drug effects (mean ± SD on drug effects questionnaire).

	Placebo	Modafinil
**Feel** ^a^	1.59 (0.88)	2.23 (1.14)
**High**	1.21 (0.52)	1.60 (0.98)
**Dislike**	1.33 (0.62)	1.43 (0.78)
**Like**	1.92 (1.18)	2.43 (1.28)
**More**	2.05 (1.32)	2.30 (1.29)

Collapsed across strategy.

a *p* = 0.035, Benjamini–Hochberg adjusted.

## Discussion

In this pre-registered study we used a parsimonious factorial design to examine the pharmacological effects of modafinil separately from the effects of a brief mindfulness training/induction, while also testing their combined effects on mindfulness-relevant outcomes. Across a variety of outcomes, we found no evidence of additive effects of modafinil and mindfulness training, as indicated by the lack of any drug × strategy interactions. However, we did find that increases in state mindfulness that have been described in some previous studies of brief mindfulness training (e.g. Tanay and Bernstein, 2013) can be mimicked using an acute pharmacological intervention. Specifically, a single dose of 200 mg modafinil caused a significant acute increase in state mindfulness prior to any behavioural training, although this pre- to post-drug (T1–T2) effect of modafinil was small relative to the subsequent T2-to-T3 effect on state mindfulness (in both drug conditions), which we attribute to the brief mindfulness or relaxation strategy-training. In addition, exploratory post-hoc analysis indicated that participants who received the single dose of modafinil in the lab session, spent 53% more time practising their assigned strategy outside of the lab, during the following week, relative to those who received placebo. In line with previous research, we also found that modafinil improved participants’ performance on sustained attention (PVT; Repantis et al., 2010). Our findings on the effects of modafinil on affect (e.g. higher positive *and* negative affect relative to placebo at T2) are also broadly consistent with previous literature on mood effects of modafinil (Taneja et al., 2007). To our knowledge, ours is the first study to examine the effects of modafinil (+ mindfulness or relaxation) on mind-wandering. Contrary to hypotheses, we found no evidence of an effect of modafinil on mind-wandering.

In contrast to the hypothesised pharmacological main effects – which were generally confirmed – there was no *differential* effect of the two strategies on state mindfulness, which increased to a similar extent in both the mindfulness and the relaxation strategy conditions. On the other hand, we did observe higher post-strategy parasympathetic activation (reflected in higher HRV) in the mindfulness conditions relative to relaxation. Although this effect was not pre-specified, increases in HRV have been proposed as a biomarker for mindful mental states (Krygier et al., 2013). We hypothesised superior performance on the sustained attention (PVT) and mind-wandering tasks following mindfulness induction relative to relaxation, but found no evidence for this. A recently published meta-analysis on the effects of mindfulness inductions on neuropsychological functioning (Gill et al., 2020) suggested that although there is some variation in estimated effect sizes on measures of attention, the upper bound (95% confidence interval) of this effect was only *g* = 0.3. It is possible therefore that our study was adequately powered for some of our modafinil-related focal hypotheses, but underpowered for other (mindfulness training-related) effects. However, it should be noted that, descriptively, faster reaction times were observed following relaxation relative to mindfulness (i.e. the descriptive effect of strategy on PVT performance was opposite to our hypothesis), suggesting that a lack of power is an unlikely explanation for our ‘null’ findings on the PVT.

The absence of differences between the relaxation and mindfulness training strategies on the main outcomes of interest (particularly state mindfulness) highlights a general challenge in experimental studies of behavioural interventions that seek to selectively and potently activate a specific therapeutic process of interest (e.g. mindful attention). The challenge particularly arises in the design of suitable control conditions, namely conditions that resemble the active intervention and are equivalently credible, but are relatively inert (e.g. do not activate the neurophysiological/neurocognitive processes underlying mindful attentional states). In the current study, we designed the mindfulness and relaxation strategy instructions to be closely-matched in terms of duration, complexity, credibility and expectancy. Although we were successful in these respects, similar increases in state mindfulness in the two strategy conditions suggest that the relaxation instructions may have been insufficiently distinct from the mindfulness instructions, resulting in both strategies equivalently activating mindfulness-like subjective states. For example, both sets of strategy instructions indicated that participants should return to employing the strategy if their attention drifted. Thus, the relaxation instructions may have unintentionally activated a form of meta-cognitive awareness that is more typical of mindfulness exercises than relaxation. In addition, expert raters evaluated the strategy instructions to be ‘moderately’ (rather than highly) distinct in terms of the subjective and physiological states they were likely to engage (see Supplemental material). These considerations suggest that future research in this area should aim to employ more distinctive control strategies, which are nonetheless designed to be equivalent to active mindfulness conditions on credibility, expectancy, etc. (e.g. affective/cognitive suppression or reappraisal; Beadman et al., 2015).

In addition to the main effects of strategy and drug, we were interested in examining the interaction between these factors, as a first step to determining if modafinil (or related drug) might have utility as a temporary adjunct to MBIs (e.g. in early phases of treatment). Such pharmacological augmentation of MBIs might be particularly relevant for people with low levels of trait mindfulness, who may find MBI-exercises especially challenging and hence may not expect to benefit from such treatments. Indeed, mindfulness-based relapse prevention for substance use disorders (for example) has lower rates of compliance relative to standard cognitive-behavioural relapse prevention (e.g. Bowen et al., 2014). In addition, patients whose cognitive resources are depleted due to high levels of rumination or worry, benefit less from MBIs (Banerjee et al., 2018), while those with impairments in executive function and inhibitory control deficits may find it difficult to adhere to MBIs (see Witkiewitz et al., 2019). Adjunctive pharmacological interventions that counteract such barriers by, for example, lowering the threshold for attaining focused attentional states, might therefore enable wider benefits of MBIs to be realised. Although we did not find any evidence for specific augmentation of mindfulness with modafinil, we believe this is still a worthwhile focus for future research (see below).

### Limitations and future research directions

This was an experimental study examining the potential of a single dose of modafinil to mimic or enhance a specific component of behavioural training in healthy volunteers. It is possible that the relatively small effect we observed with modafinil on state mindfulness and the absence of enhancement of strategy effects, reflected our recruitment of participants with intact attentional abilities. Future studies may seek to examine these effects in vulnerable participant groups with impaired capacity to engage in mindfulness training (e.g. those with high levels of rumination, worry, substance use, or poor performance on working memory or attentional tasks). Moreover, future studies might consider simulating clinical treatment more closely by, for example, using multiple sessions of training, and combining these with multiple doses of modafinil. If the apparent modafinil-induced increase in strategy-use during the one-week follow-up is reliable, this might be expected to drive a cumulative effect of modafinil on mindfulness-related outcomes (across several sessions), especially if combined with a more distinctive (relative to the control strategy) mindfulness training than used here.

We used a 2 × 2 design here and it could be argued that in the absence of ‘no-strategy’ control conditions (for both drug groups, i.e. a 3 × 2 between subjects design), effects attributed to strategy (between T2 and T3) might in fact reflect increasing intensity of modafinil effects over time. This possibility could have been tested by repeating the DEQ at T3 to determine whether effects on the ‘feel’ item (which is unrelated to mindfulness) remained stable between T2 and T3. Future studies should therefore include suitable no-strategy conditions, or more parsimoniously, at least include more regular assessments of drug effects.

The analysis for one of the main reported findings – the effects of modafinil on strategy-use during the follow-up period – was not pre-specified. Moreover, this outcome was based on retrospective estimation of number and duration of practice occasions, and may therefore be susceptible to memory biases. There are a limited number of plausible bio-psychological mechanisms for such an effect, and as such, this result should be considered provisional, pending replication. We intend to perform such a replication in the future and have pre-specified a test of the effects of modafinil on self-practice during follow-up (see https://osf.io/zrewj). This study will involve daily monitoring of practice and therefore should be less susceptible to memory biases.

## Conclusion

We show that it is possible to acutely increase state mindfulness using a pharmacological, rather than behavioural strategy. The absence of differential strategy effects reported here suggests that future studies should employ more distinctive strategy instructions. Although we found no evidence for specific augmentation of mindfulness by modafinil, methodological improvements in future studies may allow such additive/synergistic effects to be detected. Translational studies that test these combined effects in ‘sub-clinical’ participants (e.g. those with impaired attentional performance or low trait mindfulness) are likely to be particularly informative in determining whether MBIs can be supported pharmacologically with modafinil or similar drugs in clinical groups.

## Supplemental Material

sj-docx-1-jop-10.1177_0269881121991835 – Supplemental material for Stimulating meditation: a pre-registered randomised controlled experiment combining a single dose of the cognitive enhancer, modafinil, with brief mindfulness training Click here for additional data file. Supplemental material, sj-docx-1-jop-10.1177_0269881121991835 for Stimulating meditation: a pre-registered randomised controlled experiment combining a single dose of the cognitive enhancer, modafinil, with brief mindfulness training by Emily M Thomas, Tom P Freeman, Patrick Poplutz, Kane Howden, Chandni Hindocha, Michael Bloomfield and Sunjeev K Kamboj in Journal of Psychopharmacology
