# Supplementary material for: Stimulating meditation: a pre-registered randomised controlled experiment combining a single dose of the cognitive enhancer, modafinil, with brief mindfulness training
Source: J Psychopharmacol. 2021 Mar 1;35(6):621–30. doi: 10.1177/0269881121991835 (PMC8278547; doi:10.1177/0269881121991835)
Supplement: sj-docx-1-jop-10.1177_0269881121991835 – Supplemental material for Stimulating meditation: a pre-registered randomised controlled experiment combining a single dose of the cognitive enhancer, modafinil, with brief mindfulness training [file sj-docx-1-jop-10.1177_0269881121991835.docx]

**Supplementary materials**

**Methodological supplement.**

**Participants**

*Randomisation*

Participants were randomised into the four groups (placebo-relaxation, placebo-mindfulness, modafinil-relaxation or modafinil-mindfulness) using a random number generator in a manner that allowed balanced groups at n=40 and n=80 (i.e. in two rounds of randomisation). Forty non-repeating integers (1-40; 41-80), each corresponding to a participant id, were entered into a list randomiser (Random.org). The output from the randomiser - a column of 40 randomly ordered integers - was linked to ten repeats of the sequence “1, 2, 3, 4” (each number corresponded to one of the four experimental groups) in a separate column. The column of 40 random integers was then sorted from lowest to highest value, resulting in a randomisation of the associated column of repeating 1-4s.

*Sample size*

The sample size (n=80; G*Power; Faul et al., 2007) was based on the requirements to detect a small-medium sized interaction on state mindfulness in a repeated measures within-between design. Specifically, we assumed an effect size of *f*=0.175 (based on findings in Tanay and Bernstein, 2013), a correlation between repeated measure of r=0.5, power=0.8 and *α*=0.05. Of the originally recruited n=80, two participants’ data could not be included. Of these, one participant did not complete the Day 1 lab session (relaxation-placebo group) due to distress experienced during the strategy instructions; they requested the session be terminated but did not report an adverse response at T2 (i.e. in the post-drug/pre-strategy period). Another participant misreporting screening information, which came to light after testing was completed (n=1; relaxation-placebo group). Due to practical constraints, only one of these participants was replaced, resulting in a final sample of n=79 (relaxation-placebo: n=9 men, n=10 women). Other than the participant mentioned above, no other significant within-session adverse reactions to drug or strategy were reported.

*Eligibility*

Eligibility criteria were assessed during a telephone-screening interview. The inclusion criterion was age: 18-50 years old. Exclusion criteria were: presence of mental health, substance use or other relevant medical disorders, contraindications to modafinil use, pregnancy and current breastfeeding. Health status was determined using a semi-structured interview and self-declaration of the presence/absence of disorder. For the most common mental health problems (depression and anxiety) the abbreviated versions of the Patient Health Questionnaire (PHQ-2) and Generalised Anxiety Disorder Scale (GAD-2) were used to identify significant symptom levels at screening. Those scoring ≥3 on either questionnaire were excluded. This cut-off score has acceptable sensitivity (0.76 for both PHQ and GAD-2) and specificity (0.87 for PHQ-2 and 0.81 for GAD-2) for identifying cases of depression and anxiety (Manea et al., 2016; Plummer et al, 2016). Participants were also excluded if they engaged in recent regular meditation practice (within the last six months), and/or had >20 hours lifetime total meditation experience.

**Self-report measures**

*Within-session state measures*

A primary outcome was (change in) *state* mindfulness. Of the available state mindfulness measures, the 20-item State Mindfulness Scale (SMS; Tanay & Bernstein, 2013) was the most relevant for the current study as the items clearly relate to attentional processes (*awareness of* activities of the mind and body). Participants rated each SMS item (e.g. “*I noticed pleasant and unpleasant thoughts*”; “*I felt aware of what was happening inside of me*”; “*I noticed physical sensations come and go*.”) on a five-point scale in relation to their experience in the previous 10 minutes. The SMS was completed at three time-points within the experimental session: pre-drug (T1) post-drug (T2), and post-strategy (T3).

State affect was assessed using the Positive and Negative Affect Schedule (PANAS; Watson, Clark & Tellegen, 1988) which consists of two subscales listing 10 positive and 10 negative adjectives. Participants rated each adjective in relation to their *current* experience, from 1 (very slightly or not at all) to 5 (extremely). Participants completed the PANAS at T1, T2 and T3. Finally, drug related subjective effects were assessed using the Drug Effects Questionnaire (DEQ-5; Morean et al., 2013) only at T2. The DEQ consists of five items: *feel* (the effect of the drug), *high*, *dislike*, *like*, and *want more* which were rated on a 5-point scale: 1= “not at all”; 5=“extremely”.

**Drug Preparation**

In the active drug groups (modafinil-relaxation; modafinil-mindfulness), capsules containing 200 mg of modafinil (Glenmark Pharmaceuticals Europe Limited, UK) were administered orally with water. This dose was based on studies demonstrating enhanced cognitive effects after a single administration (e.g. Turner et al, 2003). Modafinil tablets were reformulated by over-encapsulation into opaque gelatine capsules with additional skimmed milk powder (Marvel, Premier Foods, UK). Each participant in the modafinil groups took two capsules (2 x 100mg). Matched placebos (placebo-relaxation; placebo-mindfulness groups) were formulated in identical opaque capsules filled only with milk powder. Placebo participants took two placebo capsules. (Re)formulation of modafinil-containing and placebo-containing capsules was performed by researchers with no involvement in data collection. Upon (re)formulation, capsules were placed in sealed envelopes labelled with participant id.

**Design of strategy training instructions**

*Refinement of early versions of instructions*

Strategy instruction (samples provided below) were based on various existing mindfulness resources (e.g. Williams & Penman, 2011) and adapted for the current study. Qualitative feedback and ratings of these instructions were obtained from four PhD-level research clinical psychologists with extensive experience and training in general behavioural therapy interventions (including mindfulness). None of these raters were active mindfulness researchers or practitioners, and none had a declared allegiance to mindfulness-based interventions. They were also blind to the study aims and had no other involvement in it. Modifications were made in response to their feedback and the final instructions were rated on a 1(“not at all”) to 7 (“completely”) scale on two questions: “To what extent are the two different sets of instructions likely to engage distinct *subjective* states” and “To what extent are the two different sets of instructions likely to engage distinct *physiological* states”. The modal scores were 4 (“moderately”) for both items. Voice-recordings of both strategy instructions were made by a single clinician, who was blind to study aims and had no other involvement in the study. This clinician was experienced in delivering both mindfulness and relaxation interventions, but had no declared allegiance to interventions incorporating either strategy.

A *sample* of each strategy are provided below. Full instructions (audio or transcript) are available from the senior author.

***Sample mindfulness instructions***

“*One technique that can calm the mind involves focusing your attention on the breath. Here, you will listen to some instructions on really becoming aware of the breath and focusing on the various sensations that might arise as you breathe. Practicing this technique may instil a sense of ease and stillness of the mind, potentially improving your ability to concentrate and manage stress.*

*Have your feet flat on the floor and your legs uncrossed. As you’re sitting, adopt a posture that allows you to be fully aware and alert. Try to straighten your back and raise your chest a little, without making yourself tense or stiff. Now, focus your attention on the area in your body where you feel the breath sensation most strongly. When breathing through the nose, people often feel the breath most vividly in or around their nostrils. Other people feel it most in their upper body as their chest rises and falls. Take a minute now, to notice where you feel your breath most strongly. [10 sec pause]. Now that you’ve identified where you feel your breath most strongly, focus your attention on that part of your body. Try to remain focused on the part of your body where you feel the breath sensation most clearly and strongly for remainder of the exercise. Other than focusing your attention on your breath, you don’t need to do anything special with how you breathe. Just notice how your breath feels to you. Notice the various physical sensation as you breathe. Try to focus on the subtleties of these sensations and notice if and how they change….Continue to focus on the sensations of your breathe.”*

Full mindfulness instructions: 786 words; duration: 8.37 min; Flesch-Kincaid grade level: 8.1.

***Sample relaxation instructions***

*“One technique that can calm the mind involves deep, regulated breathing. Here, you will listen to some instructions on effectively controlling the breath by rhythmic deep breathing, pacing your in and outbreaths so that they’re roughly the same duration. Practicing this technique may instil a sense of ease and stillness of the mind, potentially improving your ability to concentrate and manage stress.*

*Have your feet flat on the floor and your legs uncrossed. As you’re sitting, adopt a posture that allows you to be fully relaxed. Try to loosen any muscles in your body where there’s any stiffness. Ensure your posture is as comfortable as possible, releasing any tension. Now, consider how your body behaves as you breathe. Is your breathing shallow or deep? Is it quick or slow? Does your chest rise and fall as you breathe in and out or does your abdomen move as you breathe? Consider how long your in-breathe is compared to your out-breathe. Take a moment now to notice how you’re breathing. [10 sec pause]. Now that you’ve identified your pattern of breathing, see if you can change the way you breathe so that you’re breathing deeply, and from your abdomen rather than your chest. Most importantly, aim to make your in and outbreath roughly equal in duration. Breathe in deeply into your abdomen letting your stomach rise as you breathe in through the nose….and letting your stomach fall as you breathe out from your mouth. Try to get into a rhythm so that your outbreath is a similar duration to your inbreathe. To do this, count the number of seconds during your inbreathe and the number of seconds of your out-breath, and gradually make these as even as possible”.*

Full relaxation instructions: 796 words; duration: 9.46 min; Flesch-Kincaid grade level: 7.7.

**Statistical analyses: data handling** Data were checked for missing values, errors and univariate outliers (scores with standardised residuals ≥ 3). Outliers were winsorized by replacement with the highest non-outlier+1 (Tabachnick & Fidell, 2001). This applied to n=1 mind wandering (self-caught) scores, n=2 PVT reaction time scores, n=2 RMSSD values at T1, n=1 RMSSD value at T2 and n=1 RMSSD value at T3. Per group distributions were inspected for normality and considered non-normal if visual inspection and distribution parameters (skewness/standard error or kurtosis/standard error ratios >3) indicated such deviation predominated across groups. Non-normality was evident for PANAS-negative scores, and hence these were log_10_ transformed which successfully reduced skewness and/or kurtosis. Analyses and reported *F* and *p* values relate to transformed data for PANAS-negative, although for ease of interpretation, untransformed means are presented in the main results section (Fig 3B).

There were no missing values for most outcomes obtained on Day 1 (SMS, PANAS, mind wandering). However, due to equipment failure, n=6 participants had no heart rate variability data and an additional n=1 had missing HRV data at T2 and T3. In addition, due to file writing failures, n=5 PVT reaction time files were missing. Follow-up (Day 8) values for the FFMQ, DASS, SMS and PANAS were missing for n=6. Markov Chain Monte Carlo sampling was used in multiple imputation of missing values and analyses repeated on imputed data. Since effect sizes and significance values (where p<0.05) were unaffected, results from the original dataset are reported. Departures from expected integer *dfs* reflect occasional missing data.

**Supplementary results**

**Exploratory per group bivariate correlations between Day 1 outcomes.**

The association between the main Day 1 outcomes are summarised in Table S1 in the form of Pearson’s correlation coefficients between change scores (T2-T1).

|  | ∆ SMS | ∆ PAN + | ∆ PAN – | ∆ RMSSD | MW-pro | MW-self | PVT-RT | Modafinil |
| --- | --- | --- | --- | --- | --- | --- | --- | --- |
| ∆ SMS |  | 0.442  ^p=0.004^ | 0.247 | -0.376 | 0.175 | 0.373 | -0.163 |  |
| ∆ PAN + | 0.562  ^p<0.001^ |  | 0.133 | -0.079 | 0.001 | 0.015 | -0.236 |  |
| ∆ PAN – | 0.067 | 0.170 |  | 0.271 | 0.165 | 0.303 | 0.352 |  |
| ∆ RMSSD | -0.338 | -0.056 | 0.116 |  | -0.154 | -0.002 | -0.013 |  |
| MW-pro | -0.120 | 0.126 | 0.242 | 0.294 |  | 0.555  ^p<0.001^ | -0.135 |  |
| MW-self | 0.065 | 0.155 | -0.225 | 0.149 | 0.286 |  | -0.005 |  |
| PVT-RT | -0.081 | -0.034 | 0.164 | 0.151 | -0.157 | -0.264 |  |  |
| Placebo | | | | | | | |  |

**Table S1:** Correlation coefficients between change scores on subjective measures, RMSSD, and behavioural measures of mindfulness (MW-pro and MW-self and PVT-RT). Results are presented separately for modafinil (above the diagonal; darker grey) and placebo groups (below the diagonal; light grey), collapsed over strategy. Correlations were based on n=39 in the placebo group (except for RMSSD : n=34 and PVT: n=37) and n=40 in the modafinil group (except RMSSD: n=38 and PVT=37). Where results are significant at a conservative α=0.005, p values are presented. For reference, the corrected per-group α is 0.002. PAN +; PAN -=PANAS positive and negative affect scores; RMSSD=root mean square of successive (interbeat) differences; MW-pro=probe caught; MW-self=self-caught instances of mind wandering; PVT-RT=psychomotor vigilance task-reaction times.

**Descriptive statistics from analyses of follow-up data**

A summary of measures taken at Day 1 and repeated on Day 8 is presented in Table S2.

|  | **Day 1** | | | | **Day 8** | | | |
| --- | --- | --- | --- | --- | --- | --- | --- | --- |
|  | **Placebo** | | **Modafinil** | | **Placebo** | | **Modafinil** | |
|  | **Relax** | **Mindful** | **Relax** | **Mindful** | **Relax** | **Mindful** | **Relax** | **Mindful** |
|  |  |  |  |  |  |  |  |  |
| **SMS** | 47.94 (18.73) | 53.89 (15.31) | 52.11 (17.01) | 50.06 (17.01) | 61.12 (21.01) | 58.37 (17.69) | 62.42 (18.23) | 59.17 (18.78) |
| **PANAS**  **+ve** | 24.24 (8.10) | 25.58 (9.33) | 26.00 (6.82) | 26.72 (7.81) | 26.18 (9.74) | 26.21 (8.58) | 28.68 (7.42) | 25.39 (8.29) |
| **PANAS**  **-ve** | 12.29 (2.73) | 12.21 (2.55) | 14.53 (5.60) | 11.94 (1.98) | 11.88 (3.35) | 14.00 (5.12) | 14.21 (6.03) | 12.94 (4.46) |
| ***DASS**  **depress** | 8.35 (5.16) | 7.05 (5.22) | 7.16 (6.23) | 8.00 (6.47) | 9.29 (8.42) | 6.84 (5.67) | 8.00 (7.11) | 7.56 (5.25) |
| ***FFMQ** | 46.47 (6.71) | 51.53 (5.43) | 50.37 (7.10) | 49.33 (5.80) | 48.12 (4.55) | 51.16 (5.46) | 49.58 (6.50) | 48.83 (5.11) |

**Table S2:** Estimated marginal means (± SD) for outcomes assessed on Day 1 and Day 8. SMS and PANAS values on Day 1 were obtained at T1. * Day 1 values diverge from those in Table 1 in the main results section of the paper due to list-wise deletion resulting from missing values on Day 8. NB analyses of PANAS-negative scores presented in the main results section were performed on log transformed values.

**Supplementary references**

Faul F, Erdfelder E, Lang AG, et al. (2007) G* Power 3: a flexible statistical power analysis program for the social, behavioral, and biomedical sciences. *Behavioural Research Methods* 39: 175–191.

Manea L, Gilbody S, Hewitt C, North A, Plummer F, Richardson R, Thombs BD, Williams B, McMillan D (2016). Identifying depression with the PHQ-2: A diagnostic meta-analysis. *Journal of Affective Disorders*. 203: 382-95.

Plummer F, Manea L, Trepel D, McMillan D (2016). Screening for anxiety disorders with the GAD-7 and GAD-2: a systematic review and diagnostic metaanalysis. *General Hospital Psychiatry*. 39: 24-31.

Tabachnick BG and Fidell LS (2001) Using Multivariate Statistics (5^th^ ed.). Pearson: Needham Heights, MA.

Tanay G and Bernstein A (2013) State Mindfulness Scale (SMS): Development and initial validation. *Psychological Assessment* 25(4): 1286-1299.

Williams M, Penman D (2011) Mindfulness: The Eight-Week Meditation Programme for a Frantic World (M Williams, Narr.) [Audiobook]. Hatchet Audio UK
